# P-1300. Activity of Cefiderocol and Comparator Agents Against Metallo-β-Lactamase Carrying Acinetobacter baumannii-calcoaceticus complex Isolates Collected in the SENTRY Antimicrobial Surveillance Program

**DOI:** 10.1093/ofid/ofaf695.1488

**Published:** 2026-01-11

**Authors:** Boudewijn L DeJonge, Sean T Nguyen, Joshua Maher, Rodrigo E Mendes, Christopher M Longshaw, Hidenori Yamashiro, Yoshinori Yamano

**Affiliations:** Shionogi Inc., Florham Park, NJ; Shionogi Inc., Florham Park, NJ; Element Materials Technology/Jones Microbiology Institute, North Liberty, Iowa; Element Iowa City (JMI Laboratories), North Liberty, IA; Shionogi B.V., London, England, United Kingdom; Shionogi & Co., Ltd., Toyonaka, Osaka, Japan; Shionogi & Co., Ltd., Toyonaka, Osaka, Japan

## Abstract

**Background:**

*Acinetobacter baumannii-calcoaceticus* complex (*A. baumannii*) has become highly resistant to multiple antibiotics, especially isolates that carry metallo-β-lactamases (MBLs). Cefiderocol is a siderophore-conjugated cephalosporin and one of the few agents with activity against Gram-negative isolates carrying MBLs. In this study the activity of cefiderocol and comparator agents was assessed against MBL-carrying *A. baumannii* from the SENTRY program.

**Table 1:**
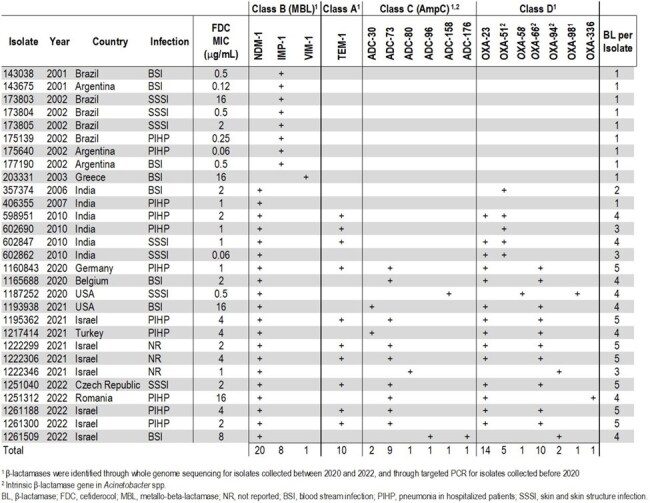
Origin, cefiderocol MIC, and β-lactamase content for clinical Acinetobacter baumannii-calcoaceticus species complex isolates tested in this study (n=29)

**Table 2:**
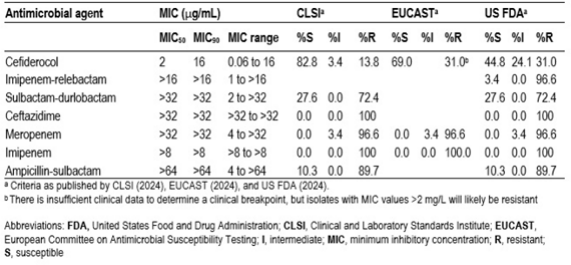
Antimicrobial activity of cefiderocol and comparator agents tested against metallo-β-lactamase-producing Acinetobacter baumannii-calcoaceticus complex isolates from the SENTRY collection (n=29)

**Methods:**

Twenty-nine clinical *A. baumannii*, collected as part of the SENTRY Antimicrobial Surveillance Program between 2001-2022, were identified as carrying MBLs. The isolates originated from at least three different infection types from eleven countries, and the majority carried NDM alleles (n=20), while eight isolates, all from Latin America, carried IMP and a single isolate from Greece carried a VIM allele (Table 1). Minimum inhibitory concentrations (MIC) for comparator agents were determined according to CLSI guidelines using broth microdilution with cation-adjusted Mueller-Hinton broth (CAMHB), whereas iron-depleted CAMHB was used for cefiderocol. Susceptibility was assessed according to 2024 CLSI, FDA, and EUCAST breakpoints.

**Results:**

MIC values for cefiderocol ranged from 0.06 to 16 µg/mL, with IMP-carrying isolates showing lower values compared to NDM-carrying isolates (Table 1). 82.8%, 69.0%, and 44.8% of the isolates tested as susceptible for cefiderocol according to CLSI, EUCAST, and FDA breakpoints, respectively, whereas β-lactam comparator agents, including sulbactam-durlabactam, all showed much lower suceptibilities (< 30%) (Table 2). NDM-carrying isolates often co-carried additional β-lactamases, but no correlation between their carriage and cefiderocol MIC value could be delineated, indicating additional factors -beyond MBL and other β-lactamase carriage- play a role in resistance to this agent in *A. baumannii*.

**Conclusion:**

Cefiderocol remained one of the few agents with in vitro activity against MBL-carrying *A. baumannii* and should be considered as a component of combination therapy when infections caused by these multidrug-resistant isolates are encountered.

**Disclosures:**

Boudewijn L. DeJonge, PhD, Shionogi Inc.: Employee Sean T. Nguyen, PharmD, Shionogi Inc: Employee Rodrigo E. Mendes, PhD, GSK: Grant/Research Support|Shionogi & Co., Ltd.: Grant/Research Support|United States Food and Drug Administration: FDA Contract Number: 75F40123C00140 Christopher M. Longshaw, PhD, Shionogi BV: Employee Hidenori Yamashiro, Shionogi HQ: Employee Yoshinori Yamano, PhD, Shionogi HQ: Employee

